# Deer antler based active ingredients have protective effects on LPS/d-GalN-induced acute liver injury in mice through MAPK and NF-κB signalling pathways

**DOI:** 10.1080/13880209.2022.2068617

**Published:** 2022-05-28

**Authors:** Guixiang He, Quanmin Zhao, Yan Zhao, Ying Zong, Shigang Gu, Mengjie Li, Renjie Li, Jiaxin Sun

**Affiliations:** College of Chinese Medicinal Materials, Jilin Agricultural University, Changchun, China

**Keywords:** RAW264.7 cells, histopathology, anti-inflammatory, antioxidant

## Abstract

**Context:**

Deer antler based active ingredients are known to have certain anti-inflammatory and antioxidant activities. However, its potential hepatoprotective effect remains unclear.

**Objective:**

This article reports the hepatoprotective effect of protein components in deer antler bases (R1) on lipopolysaccharide/d-galactosamine (LPS/d-GalN)-induced acute liver injury (ALI) in mice, and explores its possible mechanism.

**Materials and methods:**

The four separated and purified protein components of deer antler bases were screened and verified by the RAW264.7 cell inflammation model. In the *in vivo* experiment of LPS/d-GalN-induced ALI in mice, ALT, AST, SOD, CAT, GSH and MDA were detected. The liver histopathology was analysed, the COX-2 and iNOS proteins were analysed by immunohistochemistry, and 4-HNE was analysed by immunofluorescence staining. In addition, the effects on the MAPK pathway and NF-κB/IκB-α pathway in liver proteins were explored.

**Results:**

With isolated RA protein fraction pre-treated RAW264.7 cells, NO production decreased by 35.3% compared with the model group. The experimental results of ALI in mice induced by LPS/d-GalN show that R1 protein components can protect mice from ALI through anti-inflammatory and anti-oxidative stress effects and reduce liver pathological damage in mice. The results also indicate that the R1 protein component may protect the liver by inhibiting the activation of the MAPK pathway and the NF-κB/IκB-α pathway induced by LPS/d-GalN.

**Conclusions:**

The separated and purified R1 protein component of deer antler base has a good protective effect on LPS/d-GalN-induced liver injury, and may become a potential material for protecting against liver injury.

## Introduction

According to the global burden of disease statistics, more than 2 million people died of liver disease in 2010, mainly including acute hepatitis, cirrhosis and liver cancer, accounting for about 4% of global deaths (Pimpin et al. 2018). Liver disease has been shown to be one of the most serious health problems in the world. Liver injury may be caused by many factors, including hepatitis virus infection, drug toxicity, alcohol, industrial chemicals and bacterial metabolites (Sun et al. 2011).

Acute liver injury (ALI) is a sudden disease with many causes. The main pathological feature is excessive inflammation, oxidative stress-induced apoptosis and necrosis of liver cells (Tao Y-C et al. 2019). Lipopolysaccharide (LPS) is an important component of the outer membrane of Gram-negative bacteria, which can cause endotoxin damage and stimulate immune cells to release inflammatory factors such as TNF-α, IL-6 and IL-1β, thus causing apoptosis and necrosis of hepatocytes (Wirthgen et al. 2017). d-Galactosamine (d-GalN) depletes uridine phosphate through competitive inhibition, thereby inhibiting the synthesis of nucleic acid and protein, and causing liver cell damage (Ramos-Chávez et al. 2018). This eventually leads to liver inflammation and diffuse necrosis of hepatocytes, which can amplify LPS-induced liver damage (Wen et al. 2013). The combined use of LPS and d-GalN induced ALI in mice was morphologically and functionally similar to clinical hepatitis (Won-Jun et al. 2013). Therefore, lipopolysaccharide/d-galactosamine (LPS/d-GalN) induced ALI in mice is a common model in the study of the protective mechanism of drugs on the liver (Yuan et al. 2019).

NF-κB is an important classic nuclear transcription factor. The NF-κB transcription factor family plays an important role in inflammation and innate immune regulation (Baldwin 1996). When subjected to various stimuli from the external environment, IκB kinase is ubiquitinated and phosphorylated. The modified form dissociates NF-κB from IκB molecules, allowing the nuclear transcription factor NF-κB to enter the nucleus to regulate the expression of downstream related pro-inflammatory factors downstream (Katz et al. 1991; Miyamoto et al. 1995). Inhibition of the NF-κB signalling pathway helps to reduce body damage caused by oxidative stress and inflammation (Tak and Firestein 2001). In addition to NF-κB, MAPKs family proteins also play an important role in inflammation (Iida et al. 2007; He et al. 2014). Activated p38 is an essential molecule for inflammatory expression; ERK and JNK pathways play an important role in regulating inflammatory cytokines such as TNF-α, IL-1β and IL-6 (Xie et al. 2016).

The deer antler base is the ossified antler remaining on the stalk of the male sika deer after sawing off the antler. The following spring, when new antlers begin to grow, the antler base fall off on its own, so they are painless and available at a lower cost (Wu et al. 2013; Jiang et al. 2014). It is a part of deer antlers and is also an edible medicine. Its efficacy is similar to that of some other deer antlers (Wu et al. 2013). Modern medical research results prove that deer antler base has pharmacological effects such as anti-inflammatory and analgesic, anti-fatigue, improving sexual function (Zang et al. 2016), enhancing immunity, preventing osteoporosis and treating breast enlargement and mastitis (Zha et al. 2013; Hu et al. 2015; Tao W et al. 2018). Therefore, the purpose of this study is to isolate a protein component from the deer antler base, put forward the scientific hypothesis that this protein component protects mice from ALI, and study the specific mechanism of its protection of the liver.

## Materials and methods

### Materials

Deer antler base powder was purchased from Jilin Zhenyuan Deer Industry Co., Ltd. (Changchun, China) and was identified by Professor Zhao Yan of Jilin Agricultural University. Lipopolysaccharide from *Escherichia coli* O127:B8 and d-GalN were purchased from Sigma (St. Louis, MO). The aspartate transaminase (AST), alanine aminotransferase (ALT), malondialdehyde (MDA), superoxide dismutase (SOD), catalase (CAT) and glutathione (GSH) detection kits were provided by Nanjing Jiancheng Bioengineering Research Institute (Nanjing, China). The ELISA kits for tumour necrosis factor-α (TNF-α), interleukin-6 (IL-6) and interleukin-1β (IL-1β) were purchased from R&D systems (Minneapolis, MN). Antibodies used for immunohistochemistry and immunofluorescence including COX-2 (ab88522, 1:200), iNOS (ab1789451:200) and 4-HNE (ab46545, 1:100) were purchased from Abcam (Cambridge, UK). Western blot analysis of antibodies ERK (WL01770,1:750), p-ERK (WLP1512,1:750), JNK (WL01295,1:500), p-JNK (WL01813,1:500), p38 (WL00764,1:750), p-p38 (WLP1576,1:750), p-65 (WL01273b,1:750), p-p65 (WL02169,1:750), IκB-α (WL01936,1:500), p-IκB-α (WL02495,1:500) and β-actin (WL01372,1:5000) were all purchased from Shenyang Wanlei Biotechnology Co., Ltd. (Shenyang, China).

### Preparation of active ingredients in deer antler base

Two methods were used to extract the effective components of deer antler base: (1) 10 g deer antler base powder was added with 250 mL distilled water, heated in a water bath at 100 °C for 6 h and promptly replenished with distilled water, centrifuged at 3800 rpm for 20 min, the supernatant was collected, lyophilized in a freeze dryer to obtain crude protein, weighed and stored at low temperature. (2) Take 10 g of deer antler base powder, add 150 mL of 20 mM disodium hydrogen phosphate buffer (containing 0.3 M NaCl and 30 mM EDTA) with pH 9.0, place it at 4 °C, extract for 24 h, centrifuge at 3800 rpm for 20 min, repeatedly extract for three times, and combine the supernatant. Precipitate the supernatant with ammonium sulphate, add ammonium sulphate to saturation and fully dissolve it, then place it at 4 °C for 24 h, then centrifuge at 3800 rpm for 25 min, and take out the precipitated protein. Put the effective components of the deer antler base into a 3500 Da dialysis bag. Dialyse with pure water and test the dialysis degree with barium chloride until no white precipitate precipitates out of the dialysis outer solution by adding barium chloride, and freeze-dry the solution inside the dialysis bag.

### Purification of deer antler base peptides

The deer antler base protein was purified by dextran G-100 gel column. Take 0.1 g of lyophilized deer antler-based total peptide, dissolve it with 5 mL deionized water and pass it for 0.45 µm filter membrane, which is the loading solution. The sample is slowly added along the column wall with a rubber-tipped dropper and rinsed with deionized water at a flow rate of 0.5 mL/min, 3 mL per tube, and the absorbance value at 280 nm is detected with a UV spectrophotometer, and the pre-peak portion is collected and lyophilized with a freeze-dryer. The lyophilized product should be stored at −20 °C for backup.

### Cell experiment

#### Cell culture

RAW264.7 macrophages were purchased from the Chinese Academy of Sciences (Shanghai, China). The cells were cultured in DMEM supplemented with 10% foetal bovine serum, 100 U/mL penicillin and 100 µg/mL streptomycin, and placed in a constant temperature incubator containing 5% CO_2_ at 37 °C.

#### Cell viability assay

RAW264.7 cells were seeded into 96-well plates at a density of 5 × 10^3^ cells/well and incubated overnight. The cells were then treated with protein fractions of different concentrations (0.5, 2, 10, 40 and 80 µg/mL) for 24 h. After adding the MTT working solution, the cells were stained for another 4 h in the dark at 37 °C. Then discard the supernatant and add 150 µL of dimethyl sulphoxide (DMSO) to dissolve the formazan crystals. Measure the absorbance at 490 nm in a microplate reader.

#### Measurement of nitric oxide levels

RAW264.7 cells were seeded into 96-well plates at a density of 1 × 10^4^ cells/well. RAW264.7 cells were pre-incubated with protein fractions for 4 h, then LPS stimulation (1 µg/mL) was performed. After 24 h of incubation, the supernatant was collected and used to measure the NO content using the NO determination kit. Measure the absorbance at 540 nm in a microplate reader.

#### The levels of pro-inflammatory cytokines

The levels of IL-1β, IL-6 and TNF-α in cell culture supernatants were measured with an ELISA kit according to manufacturer’s instructions.

### Animals and reagents

Adult male ICR mice, weighing 20–22 g, were purchased from the Changchun Yisi Experimental Animal Technology Co., Ltd. (Changchun, China). The laboratory conditions were temperature of 25 ± 2 °C, humidity of 50–55%, 12 h light/dark cycle. The mice were acclimatized to the environment for one week before the experiments. All experiments were performed according to the animal experiment guidelines of Jilin Agricultural University. The agreement was approved by the Experimental Ethics Committee of Jilin Agricultural University.

#### Experimental design

The mice were randomly divided into five groups (eight per group). Control group: the mice by intragastric administration of an equal volume of normal saline for 10 days. LPS/d-GalN group: an equal volume of normal saline was given for 10 days in the first nine days and intraperitoneal injection of LPS (50 µg/kg) combined with d-GalN (800 mg/kg) on the 10th day. R1 (50, 100 and 150 mg/kg) group: mice were continuously given the corresponding dose of R1 for 10 days. On day 10, 1 h after R1 administration, mice were intraperitoneally injected with LPS/d-GalN. For further laboratory analysis, mouse serum and liver samples were obtained 12 h after LPS/d-GalN injection.

#### Serum biochemistry

The blood of mice was collected, and the serum was collected by centrifugation at 3000×*g* at 4 °C for 10 min. According to the manufacturer's instructions, a commercial ELISA kit was used to measure serum ALT and AST levels and TNF-α, IL-1β and IL-6 levels.

#### Liver enzyme activity

Take mouse liver specimens, wash with pre-cooled saline, homogenize, centrifuge at 4000×*g* at 4 °C for 20 min, and collect the supernatant for evaluation of the contents of SOD, CAT, GSH and MDA.

### Liver histology

#### Histopathological analysis

For histopathological analysis of the liver, fresh liver tissues were immersed in 4% paraformaldehyde for 24 h for dehydration. After 24 h, they were taken out and trimmed to an appropriate size, embedded in paraffin, and cut into 5 µm thick sections. The tissue sections were stained with haematoxylin and eosin (H&E), and the liver was examined for features of inflammatory infiltration, stem cell necrosis and hyperaemia through an optical microscope.

#### Immunohistochemical analysis

Liver sections are deparaffinized and dehydrated with a series of xylenes, ethanol and water of different concentrations. Then fix the sections with citrate antigen retrieval buffer (0.01 M, pH 6.0) for 20 min, wash three times with PBS (0.01 M, pH 7.4), and incubate with 1% foetal bovine serum (dissolved in TBS buffer) for 1 h. The serum was blocked with COX-2 (1:200) and iNOS (1:200) primary antibodies at 4 °C and incubated overnight. The next day, the serum was incubated with secondary antibodies for 30 min, and then DAB staining was performed. Re-stain with H&E. The immunohistochemical staining was observed by optical microscope analysis (Olympus bx-60, Tokyo, Japan).

#### Immunofluorescence staining analysis

Similar to the immunohistochemical staining method, the liver tissue sections of each group were randomly selected for staining. The primary antibody 4-HNE (1:100) was incubated overnight at 4 °C. The next day, the secondary antibody was incubated with DyLight488 (1:400) and SABC-Cy3 (1:200). The nucleus was stained with DAPI. Use a Leica microscope to observe the immunofluorescence staining.

### Western blot analysis

Remove liver tissue from −80 °C, and add RIPA lysate homogenate for protein extraction. BCA protein concentration determination kit (Beyotime Protein Reagent Co., Shanghai, China) was used to determine the concentration. The proteins were diluted with PBS to a uniform concentration and then denatured with high temperature treatment. Take the same amount of protein from each group, separate by 12% SDS-PAGE electrophoresis, and transfer to PVDF membrane. After 5% skim milk was blocked at room temperature for 2 h, the membrane was washed with TBS-T, and the primary antibody was incubated overnight at 4 °C. The next day, after washing the membrane with TBS-T at room temperature and incubated with secondary antibody for 2 h. Wash the PVDF membrane for ECL visualization, and then perform autoradiography. Greyscale values were analysed using image pro plus 6.

### Statistical analysis

All figures were created using GraphPad Prism 6 (La Jolla, CA) and IBM SPSS Statistics 26 (Armonk, NY). Data were expressed as the mean ± standard deviation (SD). One-way analysis of variance (ANOVA) was used for statistical analysis of data, followed by Tukey’s *post hoc* multiple comparison test and display with *F*-value. Statistical significance was defined as ^#^ or *p< 0.05, and ^##^ or **p< 0.01.

## Results

### Separation and purification of deer antler base protein

**Figure 1.**
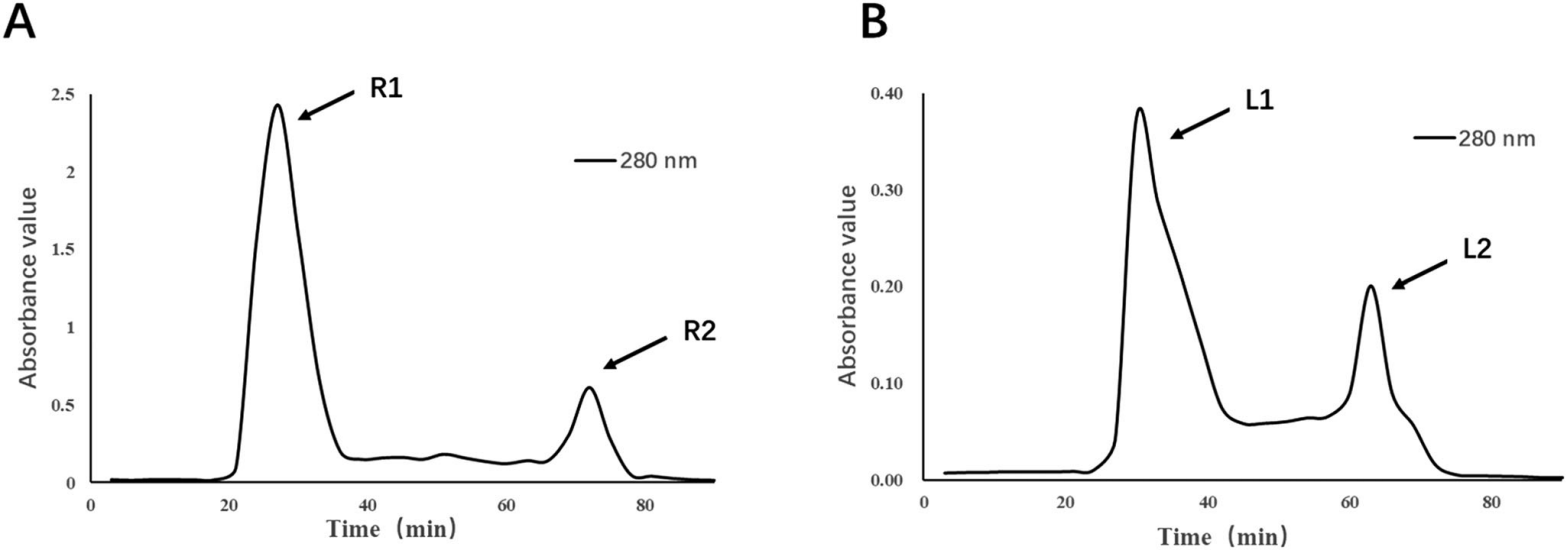
Separation and purification of crude protein from deer antler base.

The crude protein extracted thermally from distilled water and cold extracted from weak alkaline salt solution were separated and purified by Dextran G-100 gel column chromatography. Under the detection of UV spectrophotometer at 280 nm, we obtained a total of four components, R1, R2, L1 and L2 (Figure 1).

### Cytotoxicity of the separated protein

**Figure 2.**
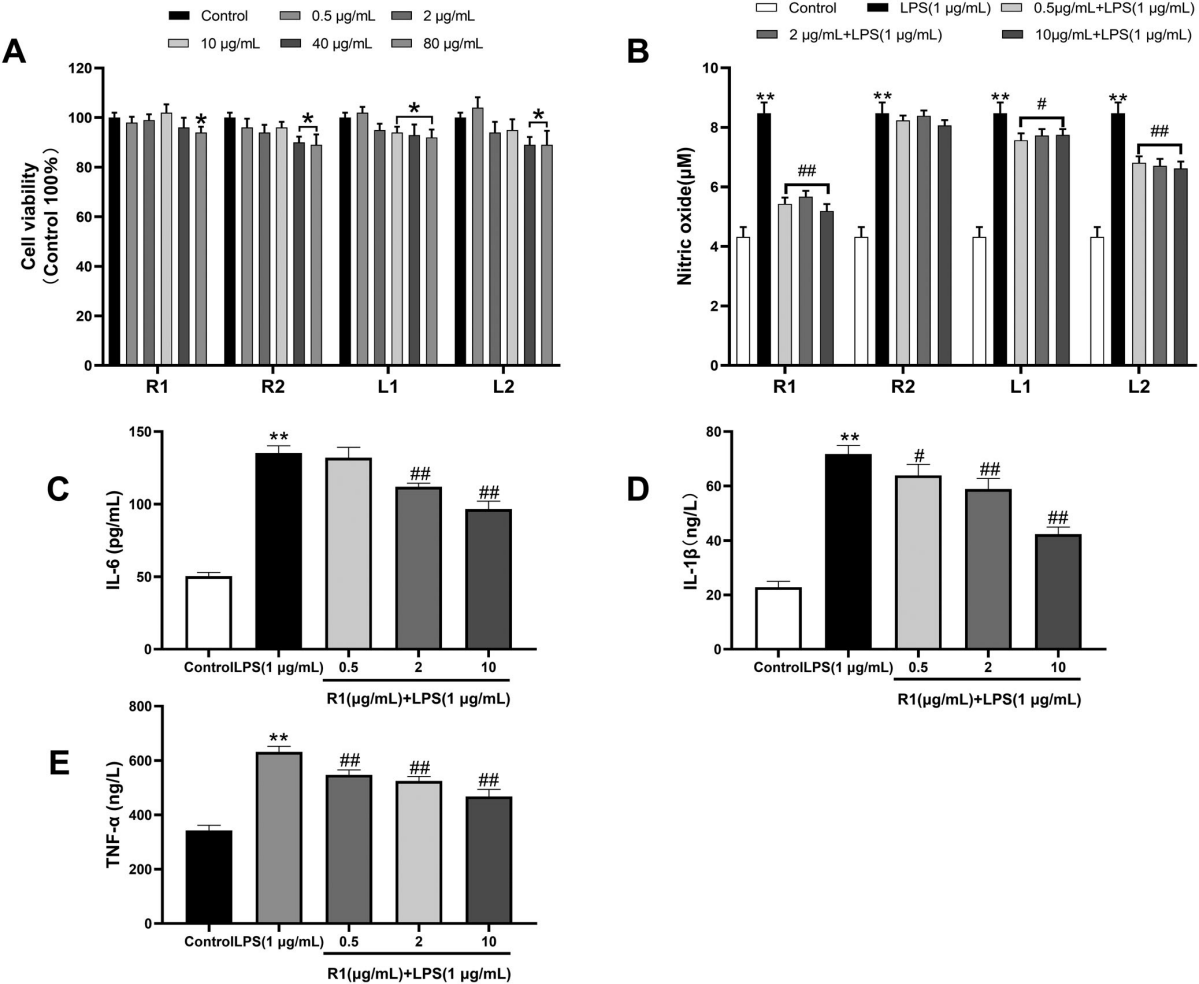
(A) The effect of different concentrations of each protein component after separation and purification on the proliferation viability of RAW264.7 cells. (B) The NO concentration produced by RAW264.7 cells induced by LPS (1 μg/mL) for 24 h and the NO produced by pre-treatment of different protein components for 4 h and then induced by LPS (1 μg/mL) for 24 h concentration. The RAW264.7 cell supernatant after pre-treatment with R1 protein components was detected with an ELISA kit to detect the levels of IL-6 (C), IL-1β (D) and TNF-α (E). Data were expressed as mean ± SD (*n* = 5 in each group). *p< 0.05, **p< 0.01 compared to the control group. ^#^*p*< 0.05 and ^##^*p*< 0.01 compared to the LPS group.

After RAW264.7 cells were incubated with each component protein at a concentration of 0.5–80 µg/mL for 24 h, the cell viability was measured by MTT method. The results showed that the cell survival rate of the four component proteins at different concentrations was higher than 90%, indicating that the separated proteins were not cytotoxic. However, the cell survival rate of R2, L1 and L2 protein at the concentration of 40 and 80 µg/mL showed a significant downward trend compared with the control group (Figure 2(A)). Therefore, in the next study, 0.5, 2 and 10 µg/mL were selected to evaluate the anti-inflammatory ability of each component protein.

### Screening for anti-inflammatory activity of isolated proteins

As shown in Figure 2(B) that the NO concentration of RAW264.7 cells induced by LPS (1 µg/mL) for 24 h was 1.97 times higher than that of the unstimulated group, and after pre-treatment of R1, R2, L1 and L2 protein components for 4 h, NO production is reduced, and R1, L1 and L2 are all significantly reduced. It is worth noting that the R1 protein component performs best in this respect, so we choose the R1 component for the next stage of research. The cytokines IL-6, IL-1β and TNF-α were measured by ELISA kit. The results shown in Figure 2(C–E) that after RAW264.7 cells were induced by LPS (1 µg/mL) for 24 h, the inflammatory factor IL-6 was significantly increased by 2.68 times compared with the control group. The inflammatory factor IL-1β was significantly increased by 3.14 times compared with the control group, and the inflammatory factor TNF-α was significantly increased by 1.85 times compared with the control group. After 4 h of pre-treatment with R1 protein components, different inflammatory factors were reduced in a dose-dependent manner.

### The effect of R1 protein component on liver ALT and AST levels

**Figure 3.**
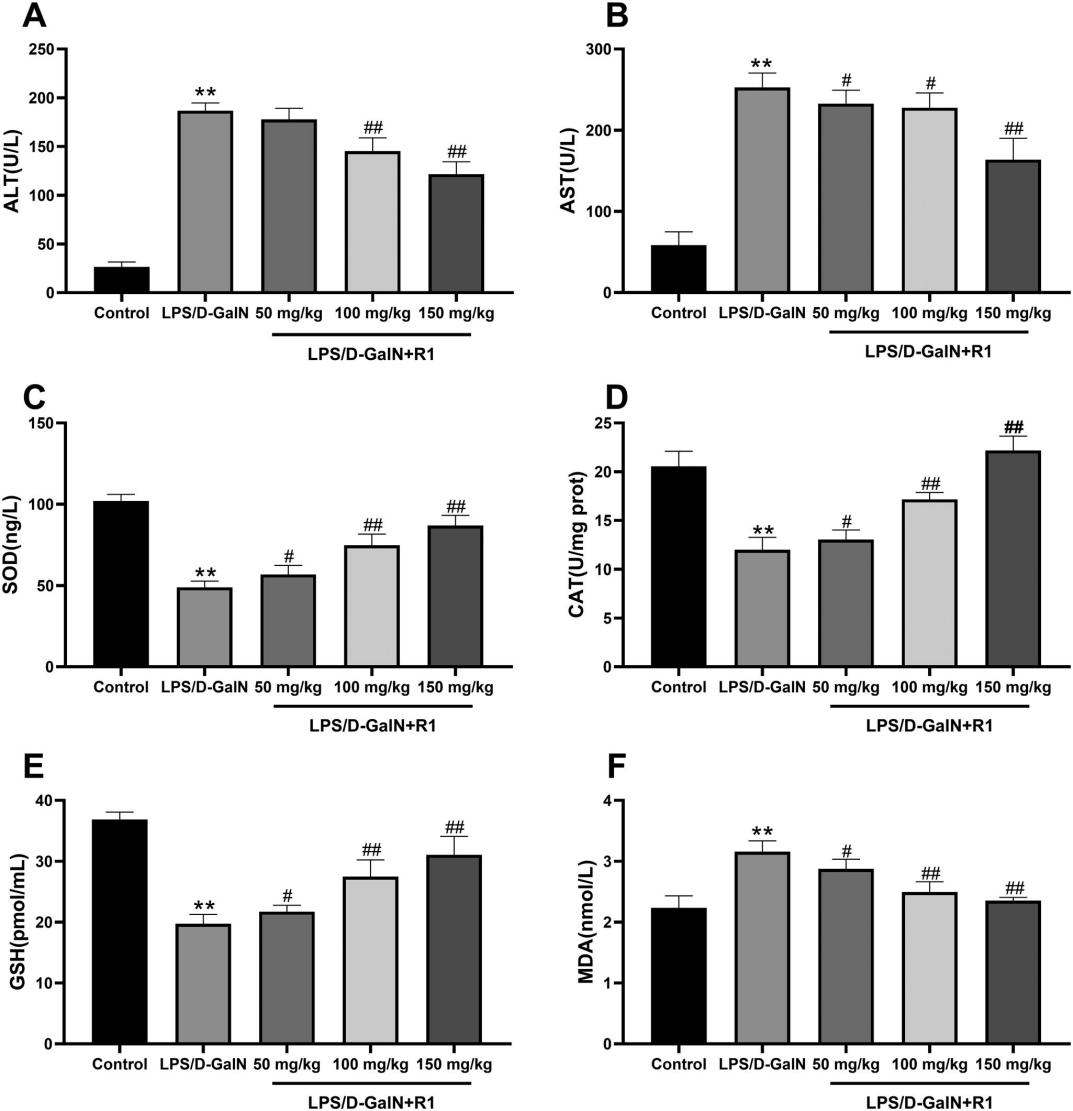
The effect of R1 protein components on ALT (A) and AST (B) in mouse serum. The effect of R1 protein components on the contents of SOD (C), CAT (D), GSH (E) and MDA (F) in the supernatant of mouse liver tissue homogenate. All data were expressed as mean ± SD (*n* = 8). **p< 0.01 compared with control group; ^#^*p*< 0.05 and ^##^*p*< 0.01 compared with LPS/d-GalN group.

Liver damage was assessed by measuring serum ALT and AST activities. As shown in Figure 3(A,B), compared with the control group, LPS/d-GalN treatment significantly increased the levels of transaminase AST and ALT (*p*< 0.01). However, R1 (50, 100 and 150 mg/kg) administration significantly attenuated this increase in a dose-dependent manner.

### The effect of R1 protein components on the levels of SOD, CAT, GSH and MDA

Malondialdehyde is the final product of the decomposition of polyunsaturated fatty acids and their related esters, and its formation is a sign of LPS/d-GalN-induced lipid peroxidation in mice. The results showed that the level of MDA caused by LPS/d-GalN exposure was significantly higher than that of the control group (*p*< 0.01). R1 (50, 100 and 150 mg/kg) pre-treatment can significantly reduce MDA caused by LPS/d-GalN exposure increase in level (*p*< 0.01). This study also measured the activities of SOD, GSH and CAT. As shown in Figure 3, the activities of SOD, GSH and CAT in the LPS/d-GalN treatment group were significantly lower than those in the control group (*p*< 0.01). Compared with the R1 (50, 100 and 150 mg/kg) pre-treatment group and the LPS/d-GalN group, the activities of SOD, GSH and CAT were significantly restored (*p*< 0.05 or *p*< 0.01).

### The effect of R1 protein component on the pathological changes of liver tissue induced by LPS/d-GalN

**Figure 4.**
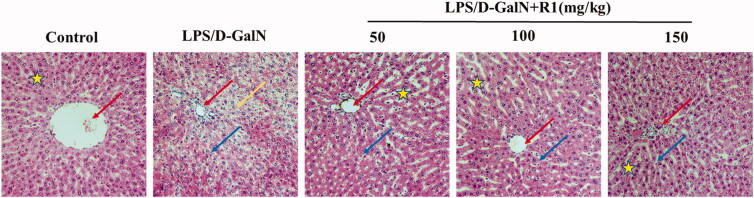
The effect of R1 protein components on the pathological changes of liver tissue. The pictures are taken from the representative histological changes of the livers of different groups of mice. Comments: central vein (red arrow), inflammatory cells (blue arrow), cellular necrosis (yellow arrow) and hepatocyte cord (pentagram).

To examine the protective effect of R1 protein components on LPS/d-GalN-induced liver injury, we observed the histopathological changes in mouse liver under light microscopy. As shown in Figure 4, the cell structure of the liver slices in the control group was normal, and the pathological changes in the LPS/d-GalN group were obvious, including extensive cell necrosis and inflammatory cell infiltration. The R1 protein component can significantly reduce the above-mentioned pathological changes.

### The R1 protein component inhibits the inflammatory response induced by LPS/d-GalN

**Figure 5.**
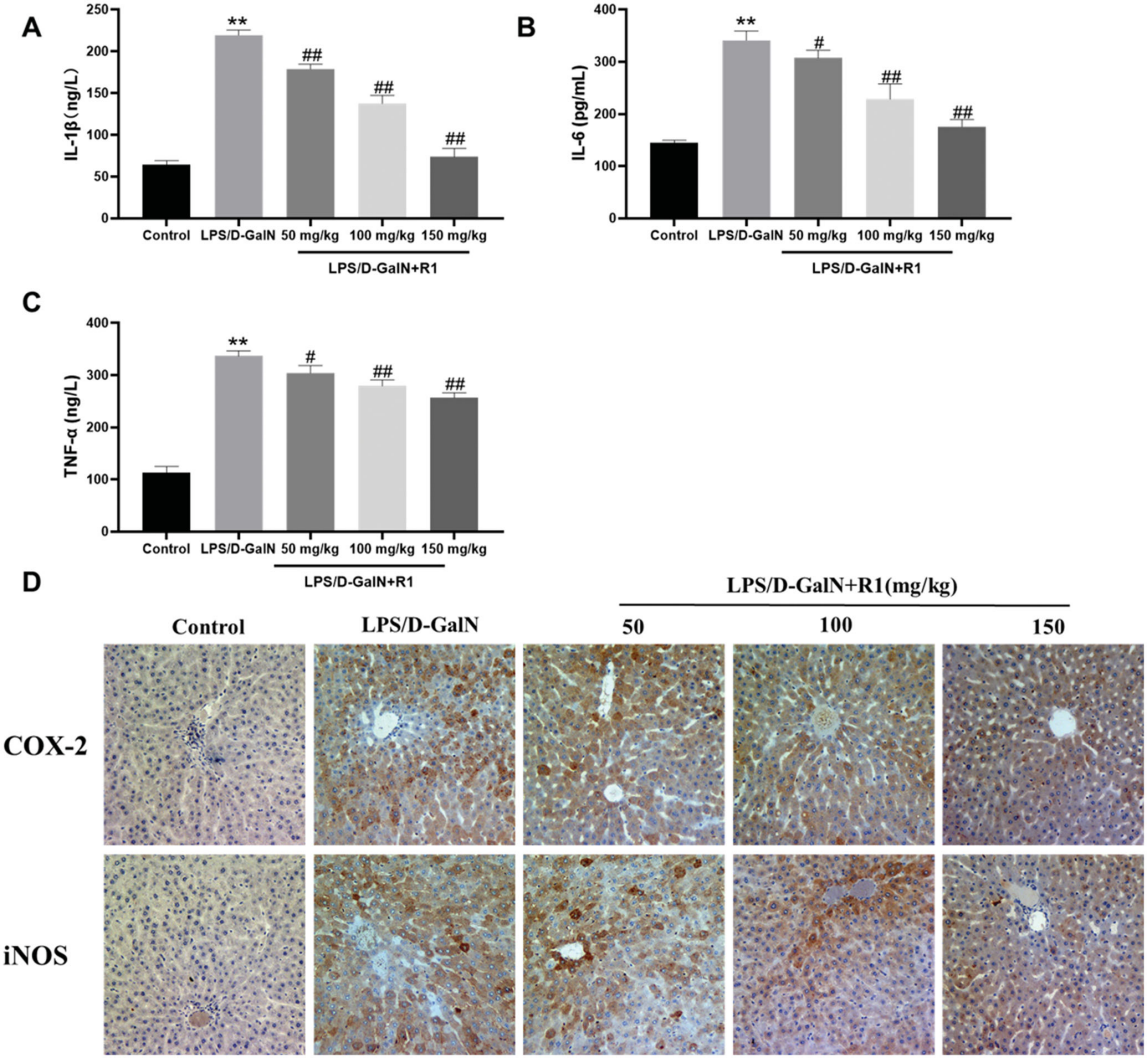
The effect of R1 protein components on the inflammatory factors IL-1β (A), IL-6 (B) and TNF-α (C) in the serum of mice. (D) The immunohistochemical staining of mouse liver, representative histological changes of livers from different groups of mice. The yellow-brown area in the figure is the positive expression part of pro-inflammatory factors COX-2 and iNOS. All data were expressed as mean ± SD (*n* = 8). **p< 0.01 compared with control group; ^#^*p*< 0.05 and ^##^*p*< 0.01 compared with LPS/d-GalN group.

Inflammatory cytokines such as TNF-α, IL-1β and IL-6 are the key pathogenic factors of LPS/d-GalN-induced acute liver failure. As shown in Figure 5(A–C), their levels in the serum of mice in the LPS/d-GalN treatment group were significantly higher than those in the control group (*p*< 0.01). Pre-administration of R1 (50, 100 and 150 mg/kg) can reduce the above increase. In addition, since the overexpression of pro-inflammatory factors iNOS and COX-2 exacerbated liver damage, we used immunohistochemistry to detect the levels of iNOS and COX-2 in liver tissue to further evaluate the anti-inflammatory response of R1. As shown in Figure 5(D), the LPS/d-GalN group in the central venous area showed a large amount of iNOS and COX-2 positive region (yellow-brown colour) expression, but this was effectively alleviated by the R1 component protein pre-treatment group. These results indicate that the R1 component protein has a hepatoprotective effect which may be related to its anti-inflammatory response.

### The R1 protein component inhibits the oxidative stress response induced by LPS/d-GalN

**Figure 6.**
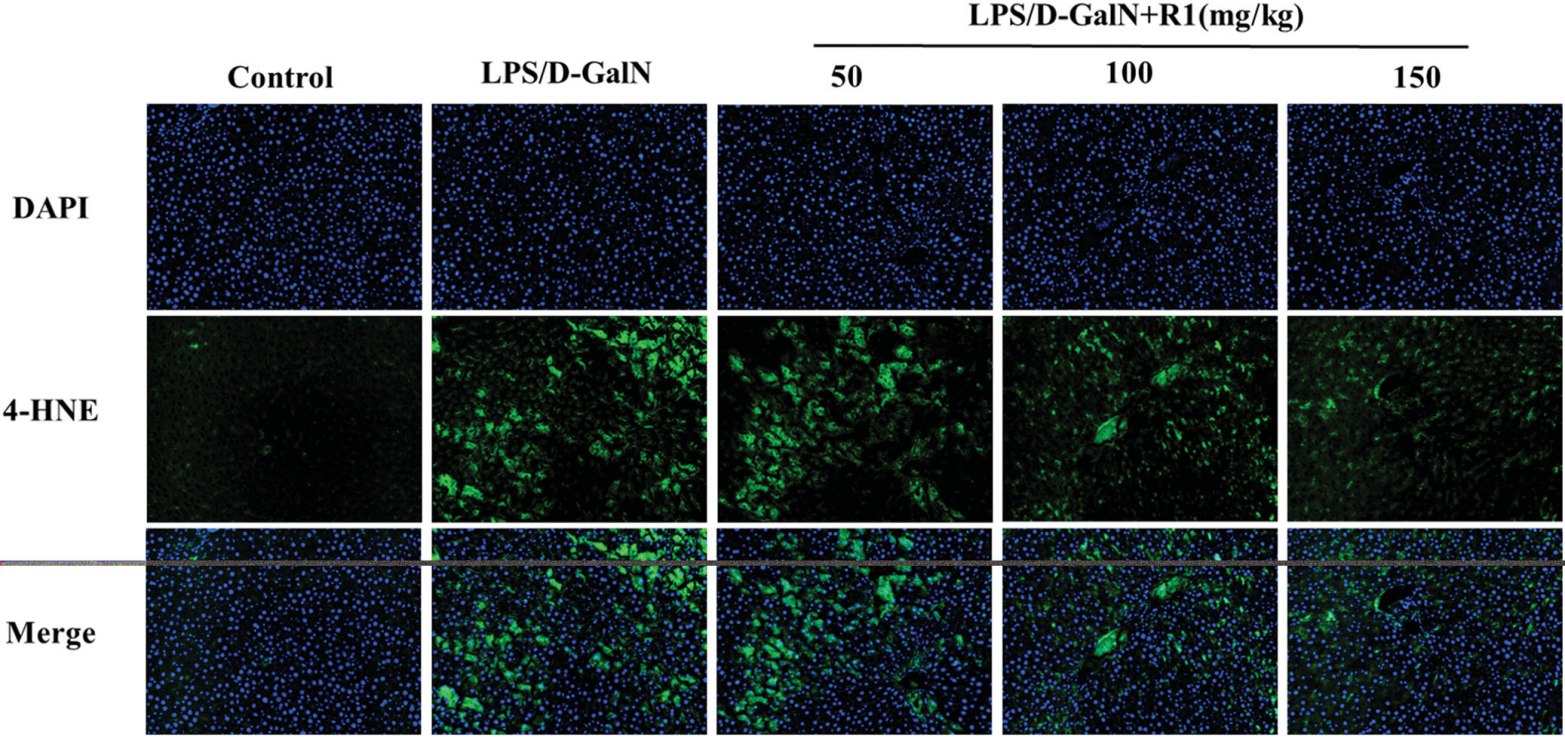
4-HNE immunofluorescence staining of mouse liver. Positive expression is strong green fluorescence, and blue indicates nuclear staining.

Oxidative stress is closely related to the molecular mechanism of LPS/d-GalN-induced ALI. As mentioned earlier, after the LPS/d-GalN model is made, the level of MDA in the liver tissue of the model group is significantly increased, and the levels of GSH and SOD are significantly reduced. In contrast, after pre-treatment with R1 (50, 100 and 150 mg/kg) for 10 days, MDA levels decreased in a dose-dependent manner and reversed the depletion of GSH and increased the activity of SOD. To further support the above conclusion, we performed immunofluorescence staining on the lipid peroxidation product 4-HNE. As shown in Figure 6, the results show that: after LPS/d-GalN modelling, the cytoplasm of the liver tissue of the model group showed strong fluorescent expression of 4-HNE. However, after 10 days of treatment with R1 (50, 100 and 150 mg/kg), the fluorescence intensity near the central vein of the liver tissue was significantly reduced and the R1 protein component reversed the expression of 4-HNE. These results indicate that the R1 protein component can at least partially alleviate the ALI induced by LPS/d-GalN by inhibiting oxidative stress.

### Effect of R1 protein components on LPS/d-GalN-induced MAPK phosphorylation

**Figure 7.**
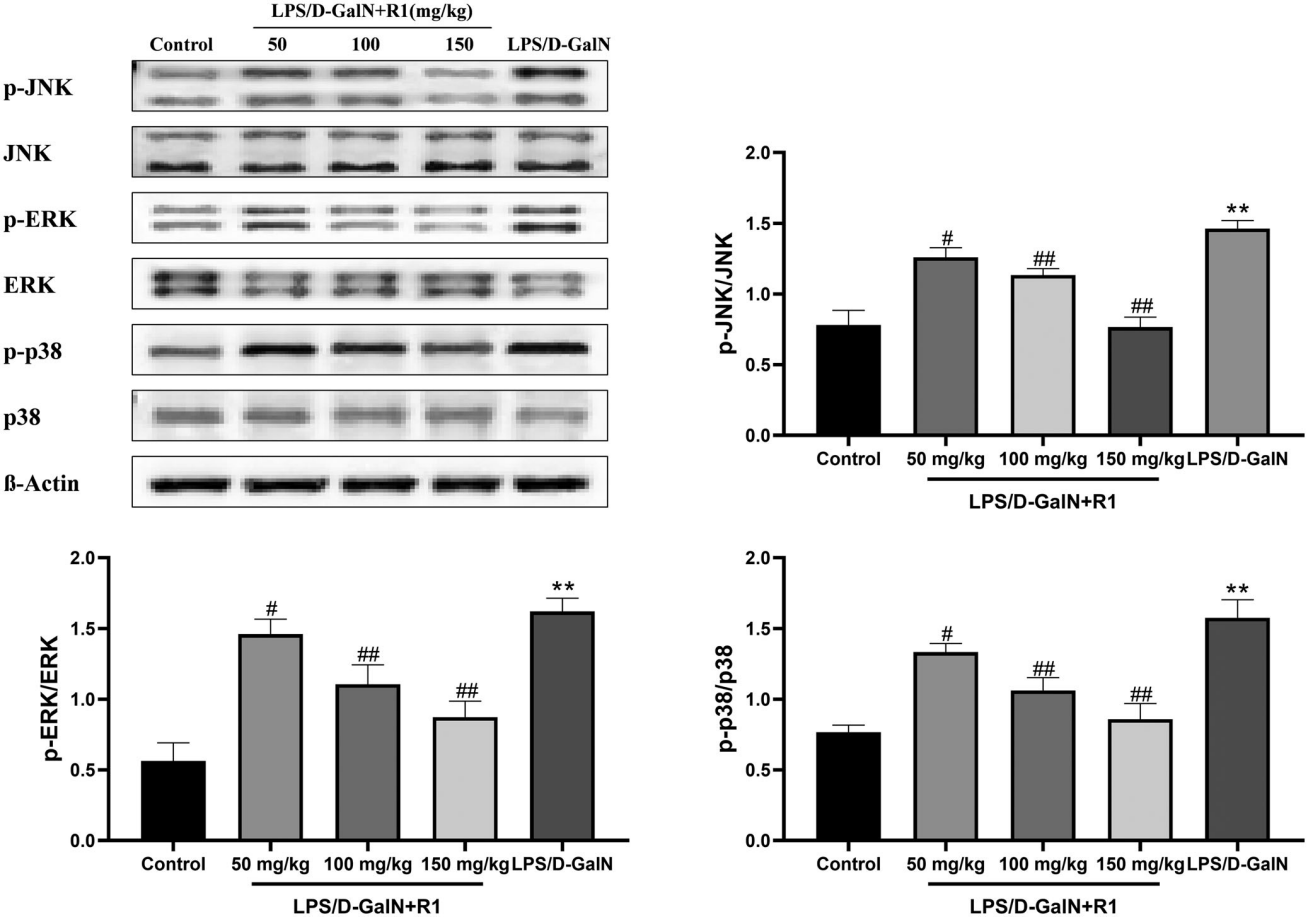
The effect of R1 protein components on MAPK phosphorylation induced by LPS/D-GalN. All data were expressed as mean ± SD (*n* = 8). **p< 0.01 compared with control group; ^#^*p*< 0.05 and ^##^*p*< 0.01 compared with LPS/d-GalN group.

In order to gain insight into the anti-inflammatory effects of R1 protein components on LPS/d-GalN-induced liver injury, we used immune protein Western blot analysis evaluated the protein level expression related to the MAPK pathway in liver tissue. As shown in Figure 7, compared with the control group, the model group mice induced by LPS/d-GalN significantly promoted the phosphorylation of ERK, JNK and p38 (*p*< 0.01). The pre-treatment of the R1 protein component effectively inhibited the phosphorylation of ERK, JNK and p38 (*p*< 0.05, *p*< 0.01). These data indicate that the R1 protein component may be activated by inhibiting MAPK activation induced by LPS/d-GalN way to protect the liver.

### Effects of R1 protein components on NF-κB pathway related proteins

**Figure 8.**
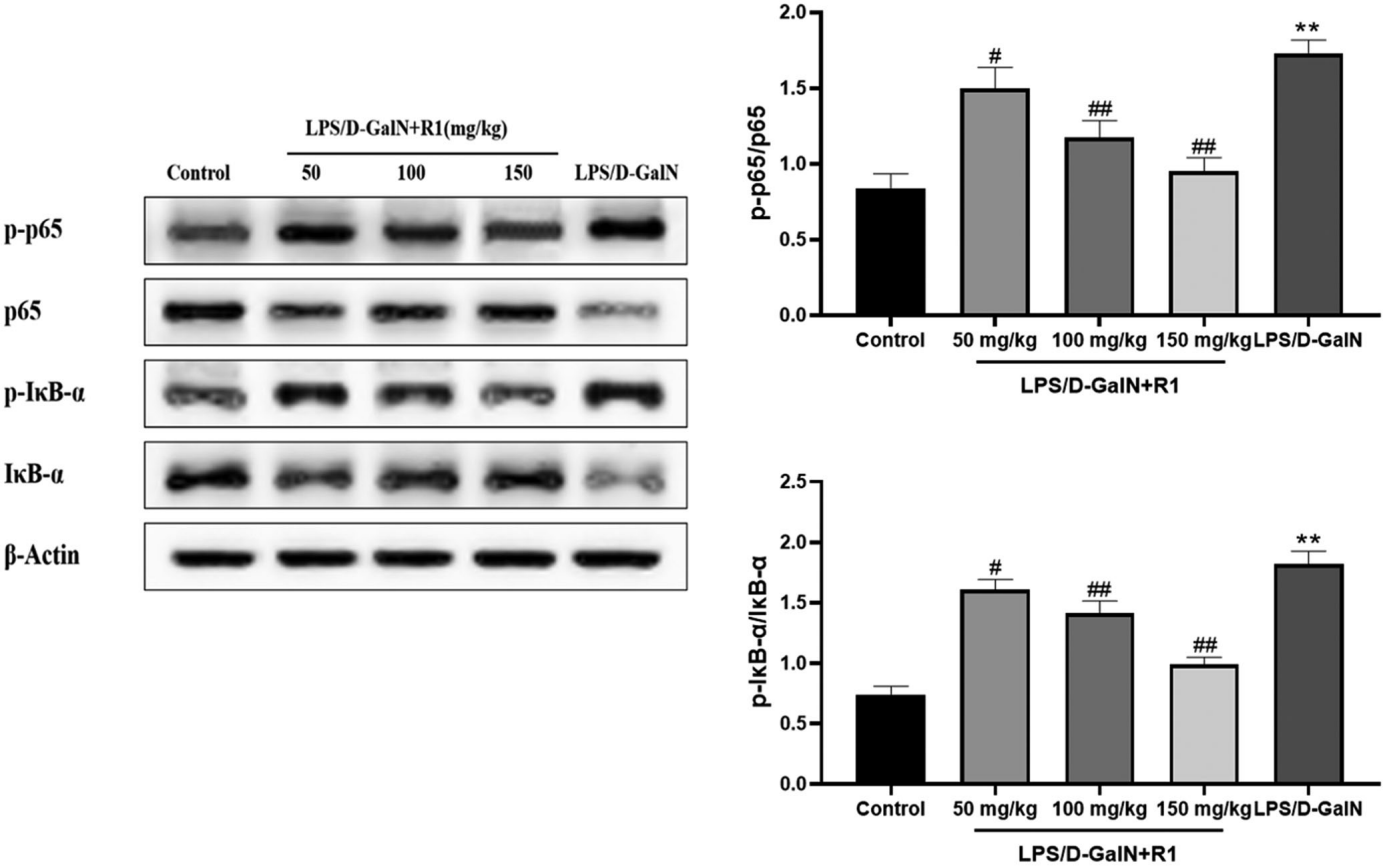
The effect of R1 protein component on LPS/d-GalN-induced NF-κB pathway related proteins. All data were expressed as mean ± SD (*n* = 8). **p < 0.01 compared with control group; ^#^*p* < 0.05 and ^##^*p* < 0.01 compared with LPS/d-GalN group.

As shown in Figure 8, compared with the control group, the model group mice induced by LPS/d-GalN significantly promoted the phosphorylation of NF-κB p65 (*p*< 0.01). The pre-treatment of R1 protein components effectively inhibited the phosphorylation of NF-κB p65 (*p*< 0.05, *p*< 0.01). Compared with the control group, the IκB-α level in the model group mice treated with LPS/d-GalN was significantly reduced, and the degradation was prevented in a dose-dependent manner after pre-treatment with the R1 protein component. On the contrary, the phosphorylation of IκB-α increased significantly after LPS/d-GalN treatment, and the R1 component protein decreased in a dose-dependent manner. These data indicate that the R1 protein component may protect the liver by inhibiting related proteins in the NF-κB pathway induced by LPS/d-GalN.

## Discussion

Acute liver injury is a serious clinical syndrome of detoxification, metabolic and synthetic dysfunction caused by severely impaired liver function (Peng et al. 2017). At present, there is no specific treatment for ALI, and emergency liver transplantation and severe simplification are often used in clinical treatment.

Macrophages are the most important immune cells and play a variety of immunomodulatory roles in various inflammatory diseases. Once activated, macrophages release a series of inflammatory cytokines (Fujiwara and Kobayashi 2005). Lipopolysaccharide is a cell wall component of Gram-negative bacteria, which interferes with the receptors of immune cells (Chen et al. 2018). Lipopolysaccharide is one of the most potent activators of mononuclear macrophages and is known to produce pro-inflammatory cytokines such as IL-1β, IL-6 and TNF-α and pro-inflammatory mediators, such as NO and PGE2 (Yang et al. 2012; Abarikwu 2014). Therefore, we selected mouse monocyte macrophages RAW 264.7 to screen the isolated and purified antler base protein extract, and found that the NO production of RAW264.7 cells after 4 h pre-treatment with R1 protein component was significantly reduced. We also measured the cytokines IL-6, IL-1β and TNF-α to further verify the anti-inflammatory effect of the R1 protein component. The results demonstrated that pre-treatment with R1 protein component, inflammatory factors were reduced in a dose-dependent manner. Therefore, we speculate that the R1 protein component of deer antler base may have a pre-protective effect on ALI through anti-inflammatory effects.

Under normal circumstances, serum ALT and AST levels are very low, but when cells are damaged, cell membrane permeability increases, and a large amount of ALT and AST enter the blood from the cytoplasm and mitochondria to increase their activity (Boll et al. 2001). Therefore, in clinical medicine, the concentration of ALT and AST in the blood is usually used as an indicator of liver function evaluation. The results of this experiment show that the R1 protein component can significantly reduce the content of ALT and AST in serum, thereby inhibiting liver damage caused by LPS/d-GalN. After H&E staining of liver histopathological sections, the model group showed a large amount of hepatocyte necrosis and inflammatory cell infiltration. However, pre-treatment of the R1 protein component significantly reduced the histopathological changes.

Many studies have shown that the main pathological characteristics of ALI induced by LPS/d-GalN are oxidative stress (Wang and Yan 2006). The content of MDA, the final product of lipid peroxidation, reflects the degree of lipid peroxidation in the liver and indirectly reflects the degree of liver cell damage (Gu et al. 2014). In the case of oxidative stress in mice, the level of MDA in the liver increases (Balasubramaniyan et al. 2003). CAT in the liver helps to remove hydrogen peroxide and avoid the production of greater hydroxyl radical toxicity. As a free radical scavenger in the body, GSH can be combined with antioxidant enzymes to prevent cell oxidative damage and death. SOD undergoes continuous oxidation and reduction of transition metal ions, and then catalyses the removal of superoxide radicals (Venukumar and Latha 2002). Due to the accumulation of superoxide free radicals and hydrogen peroxide, the reduction of these antioxidant enzymes may cause many harmful effects (Rukkumani et al. 2004). The results showed that the contents of SOD, GSH and CAT in the liver tissue of the model group treated with LPS/d-GalN decreased significantly, and the production of MDA increased significantly. After pre-treatment with R1 protein components, mice in the administration group can significantly reduce MDA levels in a dose-dependent manner, and significantly restore the activities of super SOD, GSH and CAT. In addition to the above experiments, we also performed immunofluorescence staining of the lipid peroxidation product 4-HNE in liver tissue sections. The cytoplasmic part of the liver tissues of the model group mice showed strong fluorescence expression of 4-HNE. After pre-treatment with the R1 protein component, the fluorescence intensity near the central vein of the liver tissue was significantly reduced. The above experimental results indicate that the R1 protein component protects ALI through antioxidative stress.

In addition to oxidative stress, inflammation also plays a key role in the pathogenesis of LPS/d-GalN-induced ALI. Inflammatory cytokines such as TNF-α, IL-6 and IL-1β play a key role in liver injury, and these inflammatory factors have the ability to induce hepatocyte apoptosis and necrosis (Li et al. 2014). LPS/d-GalN stimulation leads to the activation of liver macrophages/Kupffer cells, which leads to inflammatory cell infiltration and excessive production of pro-inflammatory cytokines, which ultimately leads to the formation of inflammation (Dragomir et al. 2011). The results showed that the levels of TNF-α, IL-6 and IL-1β were significantly increased in the serum of mice in the model group treated with LPS/d-GalN, while pre-treatment with R1 protein component significantly inhibited the elevation of TNF-α, IL-6 and IL-1β in the serum.

More and more studies have shown that mitogen-activated protein kinase (MAPK) and nuclear factor kappa light chain enhancer activated B cells (NF-κB) can link inflammation with many diseases (Hilliard et al. 2020). The MAPK family plays a major role in regulating the production of iNOS and pro-inflammatory cytokines. For inflammation-related diseases, most investigators have studied three parallel subfamilies of ERK, p38 and JNK (Kim et al. 2013). In general, activation of p38 is necessary for the expression of many inflammatory molecules. The ERK pathway also plays an important role in regulating NO and a variety of cytokines (including TNF-α, IL-1β and IL-6). LPS/d-GalN stimulation will cause continuous JNK activation and translocation to mitochondria, leading to increased mitochondrial permeability conversion, and ultimately leading to liver cell death (Decker and Keppler 1974; He et al. 2014). In the current study, we evaluated the effects of R1 protein components on MAPKs pathway related proteins and iNOS proteins. The results showed that LPS/d-GalN induced phosphorylation of ERK, JNK and p38, significantly inhibiting iNOS protein expression.

In addition to the MAPK protein pathway, the activation of NF-κB protein also plays a key role in the regulation of inflammation. It has been found that p65, a key protein of the NF-κB pathway, is activated and phosphorylated into the nucleus, binds to DNA, and regulates the expression of mRNAs and proteins that are downstream targets associated with inflammation or apoptosis (Quan et al. 2019). Down-regulation of p-p65 expression reduces the expression of inflammatory factors, thereby alleviating liver damage. Under normal physiological conditions, NF-κB binds to its inhibitor of NF-κB (IκB), exists in the cytoplasm as a trimer, and is in an inactive state; in TNF-α, IL-lβ and under the stimulation of LPS, etc. IκB-α will be phosphorylated and decomposed, and NF-κB will be separated from IκB-α and enter the nucleus to initiate the expression of inflammatory genes (Kern et al. 2008). The experimental results show that the pre-treatment of R1 protein components can effectively inhibit the phosphorylation of p-p65, significantly reduce the phosphorylation and decomposition of IκB-α, and play a protective effect on liver injury. Activation of the NF-κB signalling pathway will also change the expression of many inflammation-related proteins such as iNOS and COX-2. In the LPS/d-GalN-induced ALI experiment, it was found that NF-κB inhibits the expression of iNOS and COX-2 thereby reducing the inflammatory response (Tian et al. 2017). Our immunohistochemical results showed that the expression of iNOS and COX-2 in the liver pathological slices of the model group stimulated by LPS/d-GalN was significantly increased, and the R1 protein component pre-treatment group can effectively inhibit the pro-inflammatory factors iNOS and COX-2 level.

## Conclusions

The R1 protein components extracted from the antler base may protect ALI through antioxidative stress and anti-inflammatory effects, and its mechanism of action may be related to the MAPK and NF-κB protein pathways. Taken together, antler base protein may become a potential drug to prevent acute liver failure caused by LPS/d-GalN.
